# The Effects of Dietary Chromium Supplementation along with Discontinuing a High-Fat Diet on the Microbial Enzymatic Activity and the Production of SCFAs in the Faeces of Rats

**DOI:** 10.3390/nu15183962

**Published:** 2023-09-13

**Authors:** Jerzy Juśkiewicz, Katarzyna Ognik, Joanna Fotschki, Dorota Napiórkowska, Ewelina Cholewińska, Katarzyna Grzelak-Błaszczyk, Magdalena Krauze, Bartosz Fotschki

**Affiliations:** 1 Division of Food Science, Institute of Animal Reproduction and Food Research, Polish Academy of Sciences, Tuwima 10, 10-748 Olsztyn, Poland; j.fotschki@pan.olsztyn.pl (J.F.); d.napiorkowska@pan.olsztyn.pl (D.N.); b.fotschki@pan.olsztyn.pl (B.F.); 2 Department of Biochemistry and Toxicology, Faculty of Animal Sciences and Bioeconomy, University of Life Sciences in Lublin, Akademicka 13, 20-950 Lublin, Poland; katarzyna.ognik@up.lublin.pl (K.O.); ewelina.cholewinska@up.lublin.pl (E.C.); magdalena.krauze@up.lublin.pl (M.K.); 3 Institute of Food Technology and Analysis, Łódź University of Technology, Stefanowskiego 2/22, 90-537 Łódź, Poland; katarzyna.grzelak-blaszczyk@p.lodz.pl

**Keywords:** high-fat diet, dietary habits, chromium supplementation, nanoparticles, faecal microbial enzymes, SCFAs, rat

## Abstract

The present study assessed the changes in faecal microbial activity in obese Wistar rats fed high-fat or low-fat diets supplemented with various forms of chromium (picolinate or nanoparticles). The 18-week study was divided into two phases: an introductory period (9 weeks; obesity status induction via a high-fat diet) and an experimental period (9 weeks; maintained on a high-fat diet or switched to a low-fat diet and Cr supplementation). During the experimental period (10–18 weeks of feeding), samples of fresh faeces were collected on chosen days. The bacterial enzymatic activity and short-chain fatty acids (SCFAs) concentration were assessed to characterise the dynamism of the changes in faecal microbial metabolic activity under the applied dietary treatments. The results indicated that faecal microbial metabolic activity displayed several adaptation mechanisms in response to modifications in dietary conditions, and a beneficial outcome resulted from a pro-healthy dietary habit change, that is, switching from a high-fat to a low-fat diet. Dietary supplementation with chromium nanoparticles further modulated the aforementioned microbial activity, i.e., diminished the extracellular and total enzymatic activities, while the effect of chromium picolinate addition was negligible. Both the high-fat diet and the addition of chromium nanoparticles reduced SCFA concentrations and increased the faecal pH values.

## 1. Introduction

It is well known that there are changes in the intestinal microbial community under a high-fat dietary regimen and in the obesity state [1,2]. Turnbaugh et al. [3] identified the large intestinal microbiota as a contributor to the development of obesity due to an enhanced capability to metabolise dietary energetic surplus. Regardless of the health status of an organism, the large gut microbiota is capable of producing energy products in the form of short-chain fatty acids (SCFAs), but the amount of these acids and their profiles are hugely dependent on the starting substrates entering the colon/caecum and the enzymatic potential of the bacteria [4]. The main SCFAs, i.e., acetic, propionic, and butyric acids, are the products of polysaccharide and oligosaccharide microbial digestion, while proteins, peptides and glycoproteins are mainly metabolised into branched-chain fatty acids, also called putrefactive SCFAs (PSCFAs) [5]. The microbial community is able to digest the nutrients entering the large intestine due to a wide range of enzymes synthetised inside the bacterial cells and released into the intestinal environment as needed [6]. Both beneficial bacteria and those considered pathogenic have tremendous enzymatic capabilities, including the production of enzymes that can benefit or harm the host. A good example is the beta-glucuronidase produced by both *Bacteroidetes* and *Firmicutes*, the bacteria whose interactions with each other seem to be very important in obesity development [7,8]. UDP-glucuronosyltransferases conjugate hydrophobic xenobiotics and endogenous toxic substances to glucuronic acid, enhancing their solubility, and the glucuronidation process is an important detoxifying mechanism in the mammalian liver and intestinal tissue [7]. Bacterial beta-glucuronidase is able to eliminate glucuronic acid from the resultant glucuronide once it reaches the colon/caecum, allowing for the original toxic substances to be released into the gut lumen, thereby posing a threat to the health status of the body [9]. It has also been reported that the faeces of patients suffering from obesity-associated conditions (e.g., insulin resistance) are characterised by increased contents of monosaccharides, i.e., fructose, glucose, xylose, arabinose, and galactose [10]. Another study revealed that such an increase in faecal sugar levels originated from the higher activity of the faecal microbial extracellular enzymes [11]. It is well known that the gut microbiota is a contributing factor to the organism’s glucose homeostasis, metabolism, and sometimes its imbalance, and one of the paramount mechanisms is the release of glucose from complex carbohydrates (e.g., undigested, and resistant starch) due to the high α-glucosidase activity [12]. On the other hand, microbial β-glucosidase is regarded as having the ability to improve the bioavailability of polyphenolic compounds and to harvest the energy from indigestible dietary fibre [13]. In that context, microbial β-xylosidase is responsible for xylose release from some oligo- and polysaccharides that are not digested by endogenous enzymes in the small intestine, e.g., xylooligosaccharides, xylans, and arabinoxylans [14]. Therefore, in addition to the genetic analysis of the intestinal microbiota, it is important to analyse its enzymatic activity and to characterise its final metabolic products, such as SCFAs.

Some research has pointed to chromium(III) (Cr) as an active microelement in the metabolism of carbohydrates, proteins, and lipids in both humans and animals [15,16,17]. Due to these likely characteristics of Cr, publicised by some scientific reports about Cr’s capacity to control carbohydrate–lipid metabolism and decrease body weight, it is frequently utilised as a component in the supplements used in anti-obesity and other treatments [18,19,20]. Picolinate, an organic compound containing trivalent Cr, is the most widely utilised type of chromium in dietary supplements [21]. However, the existence of a relationship between chromium picolinate and either insulin resistance or type 2 diabetes is still highly uncertain [22]. Indeed, numerous studies point to major concerns about the effectiveness of Cr supplementation in the treatment of obesity and its related ailments; however, the authors do not rule out small positive health effects [23,24]. To understand the role of chromium in the treatment of diabetes and other metabolic syndrome constituents, these discrepancies warrant further extensive studies [25]. Doubts about the effectiveness of dietary Cr-picolinate are pushing researchers to look for alternative forms of supplemental chromium [26,27]. Some studies point to chromium nanoparticles as a more optimistic and effective source of this element in the diet [28]. Lien et al. [29] reported that the use of nanoparticle chromium picolinate (NanoCr-Pic) could significantly elevate Cr digestibility and serum Cr levels in rats. Our recent work showed a beneficial decrease in the caecal activity of bacterial β-glucuronidase and β-glucosidase in rats fed diets containing Cr nanoparticles compared to animals treated with dietary Cr(III)-picolinate and Cr(III)-methionine supplementation [30]. However, according to the World Health Organization and the National Institutes of Health, lifestyle modification, especially dietary changes, is the paramount strategy that may help obese patients return to a healthier, slimmer body [31]. Kalita et al. [32] revealed that negative alterations in blood and liver microelement profiles, including chromium, caused by the chronic consumption of a high-carbohydrate/high-fat diet, may be reversed upon dietary intervention. Our recent publication clearly indicates that in obese rats, the positive health-promoting effect on hepatic lipids and the inflammatory status of the liver is mainly related to the discontinuation of an unhealthy diet in favour of a low-fat diet, and chromium supplementation can only assist rather than replace such a dietary change [28].

In the present study performed on laboratory rats, it was postulated that dysbiotic variations in faecal microbial activity associated with the chronic consumption of a high-fat diet could be subsequently alleviated through the discontinuation of that dietary habit, as well as supported by supplementation with various forms of chromium. Additionally, it was hypothesised that the effects of dietary chromium nanoparticles would be more pronounced than those of the common form, namely, chromium picolinate. The faecal extracellular, intracellular, and total activities of bacterial α-glucosidase, β-glucosidase, β-glucuronidase and β-xylosidase were investigated along with the faecal short-chain fatty acid levels.

## 2. Materials and Methods

Sky Spring Nanomaterials Inc. (Houston, TX, USA) provided the Cr nanoparticles (Cr-NPs), which were characterised by a high purity (99.9% nanoparticles) and low mean size (70 nm). The common chromium source, Cr-picolinate (Cr-Pic), was purchased from Sigma (Poznań, Poland).

The National Ethics Committee for Animal Experiments approved the in vivo experimental plan (No. 73/2021). Rats (n = 84, Cmdb:Wi, male Wistar) were housed separately in cages that were kept at a constant temperature of 22 ± 1 °C and 60 ± 5% relative humidity, with 12 h day and night cycles and a ventilation air exchange of 15×h. Recently, research on hepatic metabolism has provided specifics on the experimental diet protocol [20]. In brief, the 18-week study was divided into two phases: a 9-week introductory and a 9-week experimental period. Rats were given two different diets: a low-fat (C diet) and a high-fat (F diet) diet. Furthermore, two types of supplemental dietary chromium were used: conventional Cr-picolinate and novel Cr nanoparticles (Cr-Pic and Cr-NPs, respectively), both at the pharmacologically appropriate Cr dose of 0.3 mg/kg body weight. During the second 9-week period (experimental phase), the rats were allocated into 7 groups, with 8 animals each: the low-fat C diet was consumed by Groups C and MW without the supplementation of Cr; the FW animals were subjected to the high-fat F diet without additional Cr; Groups MP and MN received additional Cr-Pic and Cr-NPs added to the C diet, respectively; and Groups FP and FN were fed the F diet with supplemental Cr-Pic and Cr-NPs, respectively. Chromium supplementation was not used during the first nine weeks of feeding (the introductory period), and the C group was given a control low-fat diet, whereas the other rats (Groups MW, FW, MP, FP, MN, and FN) were given the high-fat (F) diet to induce obesity. The dietary schema is presented in Table 1.

During the experimental period (10–18 weeks of feeding), samples of fresh faeces were collected on selected days (Days 0, 1, 3, 7, 14, 21, 48, and 63), and the faecal pH was immediately measured (pH meter, Model 301, Hanna Instruments, Vila do Conde, Portugal). Then, the samples were frozen in liquid nitrogen and stored at −70 °C until analysis. In the faecal samples, bacterial enzymatic activity and the SCFA concentration were assessed to characterise the dynamism of the changes in faecal microbial metabolic activity under the applied dietary treatments. Gas chromatography (Shimadzu GC-2010, Kyoto, Japan) was used to measure the levels of SCFAs in samples of faecal digesta (details provided in Fotschki et al. [33]). The following equipment and parameters were applied in the GC analysis: capillary column (SGE BP21, 30 m × 0.53 mm); oven temperature (initial 85 °C, final 180 °C, rate of temperature rise 8 °C/min); flame ionisation detector temperature (180 °C); and injector temperature (85 °C). Pure acetic, propionic, butyric, iso-butyric, iso-valeric, and valeric acids were purchased from Sigma Co. (Poznań, Poland). Their mixture was then utilised to generate a standard plot and to quantify the amounts of individual acids.

Bacterial enzymatic activity was measured by the rate of p-nitrophenol release from the nitrophenyl glucosides in the faecal digesta (α- and β-glucosidase, β-xylosidase, and β-glucuronidase). The following substrates were used: p-nitrophenyl-α-D-glucopyranoside (for α-glucosidase), p-nitrophenyl-β-D-glucopyranoside (for β-glucosidase), p-nitrophenyl-β-D-glucuronide (for β-glucuronidase), and p-nitrophenyl-β-D-xylopyranoside (for β-xylosidase). The p-nitrophenol concentration was quantified calorimetrically at 400 nm (details provided in Żary-Sikorska et al. [34]). The enzymatic activity was expressed as micromoles of product formed per hour per gram of faeces. The total enzymatic activity of selected enzymes, which includes intracellular and extracellular enzyme activities, was assessed after mechanical disruption of the bacterial cells by vortexing with glass beads (212–300 μm in diameter; four periods of 1 min with 1 min cooling intervals on ice) using the FastPrep^®^-24 homogeniser (MP Biomedicals, Santa Ana, CA, US). Another faecal digesta sample was not disrupted with glass beads as mentioned above, and the extracellular enzymatic activity was analysed.

### Statistical Analysis

The data were analysed using two-way ANOVA and Student’s *t* test. A single experimental group (MW, FW, MP, FP, MN, and FN) was compared with the control C group using the *t* test. Two-way ANOVA (Groups MW, FW, MP, FP, MN, and FN) was applied to assess the effects of two main factors: 1. The type of diet (D)—M (after the introductory period, the high-fat diet was stopped and the animals were switched to the control low-fat diet), F (after the introductory period, the animals remained on the high-fat diet); and 2. The dietary application of various chromium forms (W, without Cr; P, chromium picolinate; and N, chromium nanoparticles) as well as the Cr×D interaction. Duncan’s multiple range test was used to assess the means when ANOVA revealed significant treatment effects. Prior to performing any statistical analyses, the data were examined for normality. Differences with *p* ≤ 0.05 were considered significant (Statistica 12.0; StatSoft Corp., Kraków, Poland).

## 3. Results

### 3.1. The Activity of Faecal Bacterial α-Glucosidase

**Figure 1:**
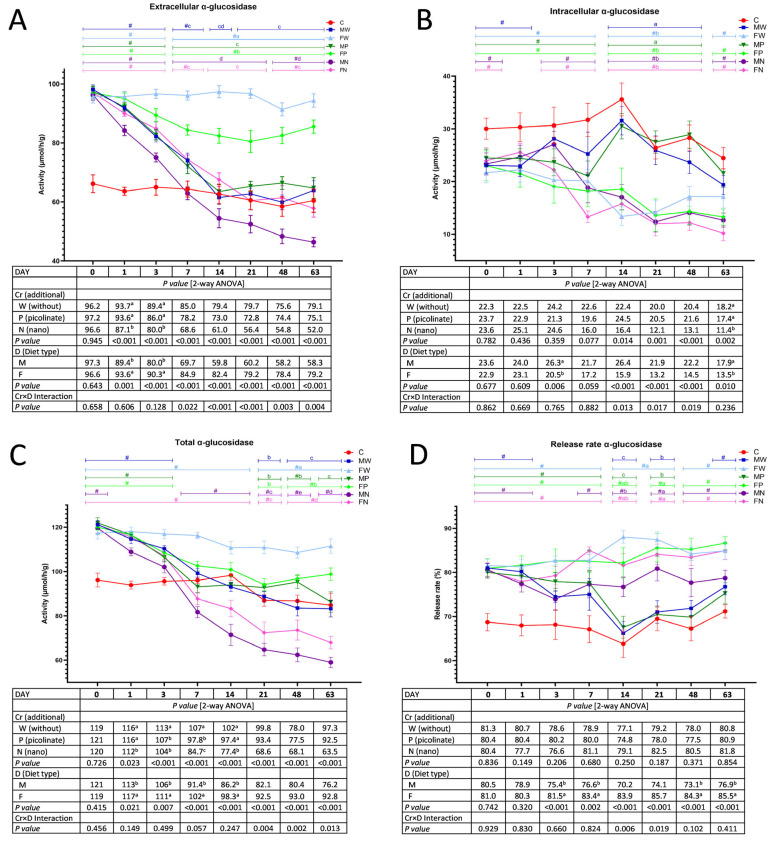
The extracellular (**A**), intracellular (**B**), total (**C**) activity and release rate (**D**) of faecal bacterial α-glucosidase during the experimental feeding period in rats (n = 8 per group). The details of the feeding periods are described in Table 1. C, animals fed a standard diet for laboratory rodents for 18 weeks; MW, animals fed the high-fat diet for 9 weeks and then the C diet for 9 weeks; FW, animals fed the high-fat diet for 18 weeks; MP, animals fed the high-fat diet for 9 weeks and then the C diet for 9 weeks with the addition of chromium picolinate; FP, animals fed the high-fat diet for 9 weeks and then the high-fat diet for 9 weeks with the addition of chromium picolinate; MN, animals fed the high-fat diet for 9 weeks and then the C diet for 9 weeks with the addition of chromium nanoparticles; FN, animals fed the high-fat diet for 9 weeks and then the high-fat diet for 9 weeks with the addition of chromium nanoparticles; M, all animals fed the high-fat diet for 9 weeks and then the C diet for 9 weeks, regardless of the type of chromium in the diet; F, all animals fed the high-fat diet for 18 weeks, regardless of the type of chromium in the diet. a–d mean values within a time point with unlike letters in the upper part of the figure are significantly different (*p* < 0.05). Only in the case of a statistically significant Cr×D interaction are the differences between groups highlighted with letters in the figure (two-way ANOVA, *p* < 0.05). # indicates a significant difference vs. the C group (*t* test, *p* < 0.05).

The two-way ANOVA showed that, irrespective of Cr dietary addition, the extracellular activity of faecal bacterial α-glucosidase was significantly higher in the F treatment versus the M treatment on Days 1 and 3 (Figure 1A). On those days, the dietary application of chromium nanoparticles caused a reduction in extracellular α-glucosidase activity in comparison to the treatments without Cr and with Cr-Pic, regardless of the diet type. The Cr×D interaction indicated that on subsequent feeding days (7, 14, 21, 48, and 63), the switch from a high-fat to a low-fat diet considerably lowered the extracellular activity of bacterial α-glucosidase, and the dietary application of Cr-NPs exacerbated that effect. The effect of dietary Cr-Pic was not significant. It should be stressed that the extracellular activity of that bacterial enzyme in the FN group (high-fat diet with Cr-NP supplementation) measured on Days 7, 14, 21, 48, and 63 was at the same level as that observed in the MW group (low-fat diet without Cr). The faeces of rats subjected to the high-fat diet during the introductory feeding period (weeks 1–9) were characterised by enhanced microbial extracellular α-glucosidase activity (*p* < 0.05 vs. C). When the *t* test analyses were considered, the rats that switched from the high-fat diet consumed in the introductory period to the low-fat diet in the experimental period had comparable α-glucosidase extracellular activity to that noted in the control C animals. This activity in group MN on Day 48 as well as in groups MN and FN on Day 63 was even lower than that in the control C rats.

Irrespective of dietary Cr addition, on Days 3 and 63, the rats fed diet F had significantly lower intracellular activity of bacterial α-glucosidase in the faeces in comparison to the M treatments (Figure 1B). On Day 63, the treatment with Cr-NPs caused a significant decrease in that activity compared to that with treatments W and P, regardless of the diet type. The nature of the significant Cr×D interaction on Days 14, 21, and 48 was that the M and MP groups exceeded the faecal bacterial intracellular α-glucosidase activity of the other groups (*p* < 0.05). Compared to the C group, the rats fed the F diet in the introductory weeks had lower intracellular α-glucosidase activity (*p* < 0.05; *t* test). The subsequent discontinuation of a high-fat diet in favour of a low-fat diet caused an increase in that activity in Group M (from the third day to the end of the study) and in Group MP (from the 14th day to the end of the experiment). As indicated in Figure 1C, the continuation of F diet consumption on Days 1, 3, 7, and 14 caused higher total bacterial α-glucosidase activity in the faeces of rats (*p* < 0.05 vs. M treatment), irrespective of dietary chromium application. On those days, regardless of the diet type, the lowest total α-glucosidase activity was noted in the group treated with Cr-NPs (on Days 1, 3, 7, and 14, *p* < 0.05 vs. W treatment; *p* < 0.05 vs. P treatment on Days 1, 7, and 14). The significant Cr×D interaction on Days 21, 48 and 63 revealed that the lowest total activity of bacterial faecal α-glucosidase was observed in both groups fed diets supplemented with chromium nanoparticles (groups MN and FN; *p* < 0.05 vs. the other groups), while the highest activity was noted in the FW rats (*p* < 0.05 vs. all the remaining groups). The *t* test showed a significantly higher total activity of bacterial faecal α-glucosidase following high-fat diet consumption in comparison to that in the feeding regimen with the control low-fat diet (C < all other groups on Day 0; *p* < 0.05). The *t* test showed that the discontinuation of the F diet in favour of a C diet normalised the total activity of that enzyme, i.e., it lowered the total α-glucosidase activity. Additionally, from Day 7 until the end of the study, the MN and FN rats had significantly lower total α-glucosidase activity in their faeces than that in the control C animals (*p* < 0.05).

Regardless of the Cr addition, the discontinuation of high-fat feeding in the experimental period resulted in a decrease in the faecal α-glucosidase release rate on Days 3, 7, 48, and 63 (M < F; *p* < 0.05; Figure 1D). On Days 14 and 21, the Cr×D interaction showed a lower percentage of the faecal α-glucosidase release rate in MW and MP rats in comparison to the remaining groups (*p* < 0.05). The *t* test showed that the release rate of that enzyme was normalised to the control C group level in the MW rats (Days 3, 7, 14, 21, and 48) and in the MP rats (from Day 14 until the end of the experiment).

### 3.2. The Activity of Faecal Bacterial β-Glucosidase

**Figure 2:**
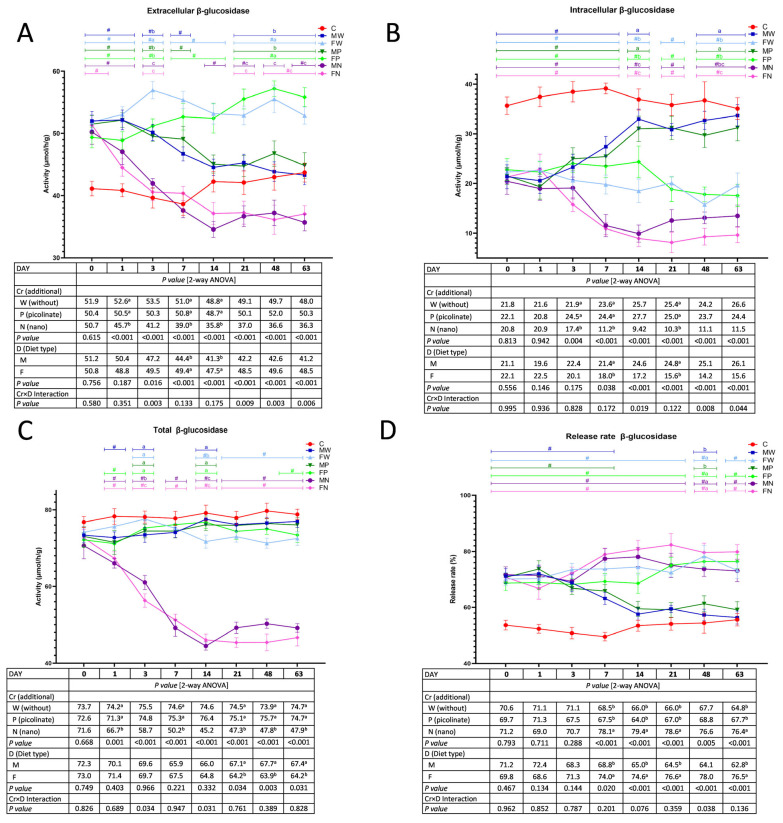
The extracellular (**A**), intracellular (**B**), total (**C**) activity and release rate (**D**) of faecal bacterial β-glucosidase during the experimental feeding period in rats (n = 8 per group). The details of the feeding periods are described in Table 1. C, animals fed a standard diet for laboratory rodents for 18 weeks; MW, animals fed the high-fat diet for 9 weeks and then the C diet for 9 weeks; FW, animals fed the high-fat diet for 18 weeks; MP, animals fed the high-fat diet for 9 weeks and then the C diet for 9 weeks with the addition of chromium picolinate; FP, animals fed the high-fat diet for 9 weeks and then the high-fat diet for 9 weeks with the addition of chromium picolinate; MN, animals fed the high-fat diet for 9 weeks and then the C diet for 9 weeks with the addition of chromium nanoparticles; FN, animals fed the high-fat diet for 9 weeks and then the high-fat diet for 9 weeks with the addition of chromium nanoparticles; M, all animals fed the high-fat diet for 9 weeks and then the C diet for 9 weeks, regardless of the type of chromium in the diet; F, all animals fed the high-fat diet for 18 weeks, regardless of the type of chromium in the diet. a–c mean values within a time point with unlike letters in the upper part of the figure are significantly different (*p* < 0.05). Only in the case of a statistically significant Cr×D interaction are the differences between groups highlighted with letters in the figure (two-way ANOVA, *p* < 0.05). # indicates a significant difference vs. the C group (*t* test, *p* < 0.05).

Irrespective of the diet type, on Days 1, 7, and 14, a significant decrease in bacterial faecal extracellular β-glucosidase activity followed the treatments with Cr-NPs versus the W and P treatments (Figure 2A). On Days 7 and 14, the two-way ANOVA showed lower extracellular β-glucosidase activity in the M treatment than in the F treatment, regardless of Cr supplementation (*p* < 0.05). A significant Cr×D interaction was noted on Days 3, 21, 48, and 63. Generally, Cr×D showed the lowest activity in both groups fed diets with chromium nanoparticles (*p* < 0.05 compared to all other groups) and the highest activity in the FW rats (*p* < 0.05 vs. all the other groups on Day 3 and *p* < 0.05 vs. all groups except FP on Days 21, 48, and 63). The *t* test showed that the addition of chromium nanoparticles to the diet had the strongest effect in reducing the enzyme activity to the level recorded in Group C (first week of the experimental part) and even below this level (from Day 14 onwards). The MW and MP groups were characterised by extracellular β-glucosidase activity comparable to that noted in Group C on Days 14, 21, 48, and 63 (*t* test, *p* > 0.05). As shown in Figure 2B, a significant decrease in the faecal intracellular activity of bacterial β-glucosidase followed dietary supplementation with chromium nanoparticles on Days 3, 7, and 21 compared to the treatments without Cr and with Cr-Pic, regardless of the diet type. On Days 7 and 21, the discontinuation of high-fat diet consumption resulted in enhanced intracellular activity of β-glucosidase, irrespective of Cr addition (*p* < 0.05 vs. F treatment). A Cr×D interaction showed that on Days 14, 48, and 63, Groups F and FP surpassed the remaining animals with respect to the abovementioned activity. Additionally, on Day 14, both groups fed Cr-NPs had the lowest activity of intracellular faecal β-glucosidase (*p* < 0.05 vs. the remaining groups), while on Days 48 and 63, the lowest activity was attributed to the FN rats (*p* < 0.05 vs. the MW, FW, MP, and FP groups). The *t* test revealed that the consumption of the high-fat diet in the introductory period resulted in decreased intracellular β-glucosidase activity (*p* < 0.05 vs. C). From Day 14 until the end of the experimental period, the activity was normalised to the C group level in rats from the MW and MP groups.

The total activity of faecal bacterial β-glucosidase was significantly diminished on Days 1, 7, 21, 48, and 63 by dietary Cr-NPs, irrespective of dietary type (*p* < 0.05 vs. the W and P treatments; Figure 2C). The switch away from a high-fat diet to a low-fat diet resulted in the elevated total activity of that enzyme on Days 21, 48, and 63, regardless of Cr addition. The nature of the significant Cr×D interaction on Days 3 and 14 indicated that the lowest activity of total β-glucosidase followed the dietary addition of chromium nanoparticles (*p* < 0.05 vs. all the other groups). Additionally, on Day 3, the FN rats had lower activity of total β-glucosidase in the faeces in comparison to that in the MN animals. Generally, the *t* test analyses (i.e., comparisons of each experimental group versus the control C group) showed that the dietary supplementation with chromium nanoparticles caused a depletion of total β-glucosidase activity from Day 1 until the end of the experimental feeding. Additionally, a significant decrease was also noted in the F rats on Days 14, 21, 48, and 63, but to a lesser extent. The discontinuation of a high-fat diet in the experimental period resulted in a significant decrease in the release rate percentage of faecal β-glucosidase on Days 7, 14, 21, and 63, regardless of Cr supplementation (*p* < 0.05 vs. F; Figure 2D). On those days, irrespective of the diet type, the Cr-NPs caused a significant increase in the faecal microbial β-glucosidase release rate in comparison to that with the W and P treatments (*p* < 0.05). The significant Cr×D interaction on Day 48 revealed a decreased faecal β-glucosidase release rate in Groups MW and MP in relation to all the other groups. The *t* test showed that, in comparison to the C rats, all groups during the experimental period had a significantly lower release rate of that enzyme except the MW and MP groups from Day 14 to the end of the study.

### 3.3. The Activity of Faecal Bacterial β-Glucuronidase

**Figure 3:**
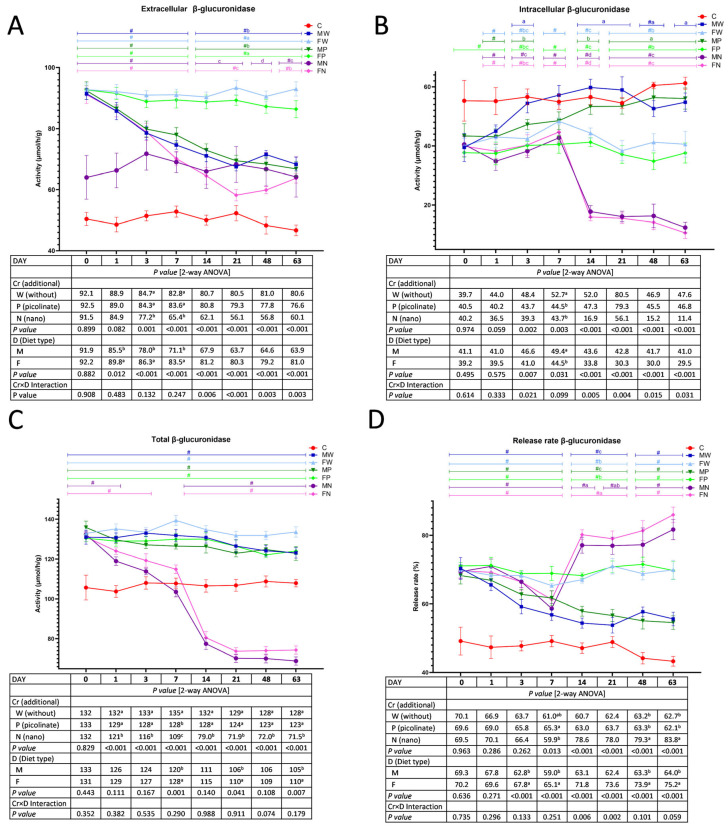
The extracellular (**A**), intracellular (**B**), total (**C**) activity and release rate (**D**) of faecal bacterial β-glucuronidase during the experimental feeding period in rats (n = 8 per group). The details of the feeding periods are described in Table 1. C, animals fed a standard diet for laboratory rodents for 18 weeks; MW, animals fed the high-fat diet for 9 weeks and then the C diet for 9 weeks; FW, animals fed the high-fat diet for 18 weeks; MP, animals fed the high-fat diet for 9 weeks and then the C diet for 9 weeks with the addition of chromium picolinate; FP, animals fed the high-fat diet for 9 weeks and then the high-fat diet for 9 weeks with the addition of chromium picolinate; MN, animals fed the high-fat diet for 9 weeks and then the C diet for 9 weeks with the addition of chromium nanoparticles; FN, animals fed the high-fat diet for 9 weeks and then the high-fat diet for 9 weeks with the addition of chromium nanoparticles; M, all animals fed the high-fat diet for 9 weeks and then the C diet for 9 weeks, regardless of the type of chromium in the diet; F, all animals fed the high-fat diet for 18 weeks, regardless of the type of chromium in the diet. a–d mean values within a time point with unlike letters in the upper part of the figure are significantly different (*p* < 0.05). Only in the case of a statistically significant Cr×D interaction are the differences between groups highlighted with letters in the figure (two-way ANOVA, *p* < 0.05). # indicates a significant difference vs. the C group (*t* test, *p* < 0.05).

Regardless of the dietary chromium addition, the discontinuation of high-fat consumption resulted in diminished activity of extracellular bacterial β-glucuronidase in the faeces of rats on Days 1, 3, and 7 (*p* < 0.05 vs. F treatment; Figure 3A). On Days 3 and 7, the faeces of rats fed diets containing chromium nanoparticles had the lowest extracellular activity of that enzyme, irrespective of the diet type (*p* < 0.05 vs. the W and P treatments). From Day 14 until the end of the experimental period, the significant Cr×D interaction indicated decreased extracellular activity of faecal β-glucuronidase in the groups fed a low-fat diet (versus the FW and FP groups), but the lowest activity was noted in the groups fed a diet containing chromium nanoparticles, especially in the MN rats. The *t* test showed that, when compared to the control C group (fed a low-fat diet during the entire 18 weeks, i.e., introductory and experimental periods), all groups during the experimental weeks had increased extracellular activity of faecal β-glucuronidase, except the MN group on Days 14, 21 and 48. The two-way ANOVA showed that, irrespective of the chromium addition, the M treatment exceeded the F treatment with respect to the intracellular β-glucuronidase activity in the faeces on Day 7 (Figure 3B). On that day, regardless of the diet type, Cr-Pic and Cr-NP supplementation resulted in diminished intracellular faecal β-glucuronidase activity (*p* < 0.05 vs. W treatment). The Cr×D interaction was noted on Days 3, 14, 21, 48 and 63. On Day 3, the highest activity of intracellular β-glucuronidase was noted in the MW rats (*p* < 0.05 vs. all other groups), while the lowest activity was noted in the MN rats (*p* < 0.05 vs. the MW and MP groups). From Day 14 to the end of the experimental feeding, the MW and MP groups had enhanced activity of microbial intracellular β-glucuronidase compared to that in the rats fed a high-fat diet without the chromium addition (Group FW) and with chromium picolinate (Group FP). In addition, the lowest activity was observed in both groups fed diets supplemented with chromium nanoparticles (Groups MN and FN). Generally, during the 10–18-week period, compared to the control C group (test-t), the rats fed the experimental diet had lower faecal activity of intracellular β-glucuronidase, except the MW and MP animals.

During the entire experimental period, the total activity of faecal microbial β-glucuronidase was significantly decreased in rats fed a diet containing chromium nanoparticles compared to the treatments without Cr and with Cr-Pic, regardless of the diet type (Figure 3C). On Days 7, 21, and 63, the switch away from a high-fat diet to a low-fat dietary regimen resulted in diminished total activity of bacterial β-glucuronidase, irrespective of Cr supplementation (F treatment>M treatment; *p* < 0.05). The *t* test showed that, in comparison to the C animals, the dietary application of chromium nanoparticles caused a decrease in the faecal total activity of bacterial β-glucuronidase, especially from Day 14 to the end of the study. The remaining groups (MW, FW, MP, and FP) had significantly higher activity during the experimental period of 10–18 weeks (*p* < 0.05 vs. C; *t* test). Two-way ANOVA showed that, irrespective of chromium supplementation, the M treatment was characterised by a decrease in the bacterial β-glucuronidase release rate percentage on Days 3, 7, 48, and 63 (*p* < 0.05 vs. F treatment; Figure 3D). Regardless of the diet type, on Day 7, the highest β-glucuronidase release rate was noted in the P treatment (*p* < 0.05 vs. N treatment), and on Days 48 and 63, the highest release rate was in the N treatment (*p* < 0.05 vs. the W and P treatments). The Cr×D interaction showed that on Days 14 and 21, the lowest β-glucuronidase release rate percentages were in MW and MP rats (*p* < 0.05 vs. all other groups), while the highest values were noted in rats fed diets containing chromium nanoparticles. The *t* test revealed that, during the entire experimental period (10–18 weeks), all groups were characterised by a higher release rate of β-glucuronidase from bacterial cells into the faecal digesta environment in comparison to that in the control C group (*p* < 0.05).

### 3.4. The Activity of Faecal Bacterial β-Xylosidase

**Figure 4:**
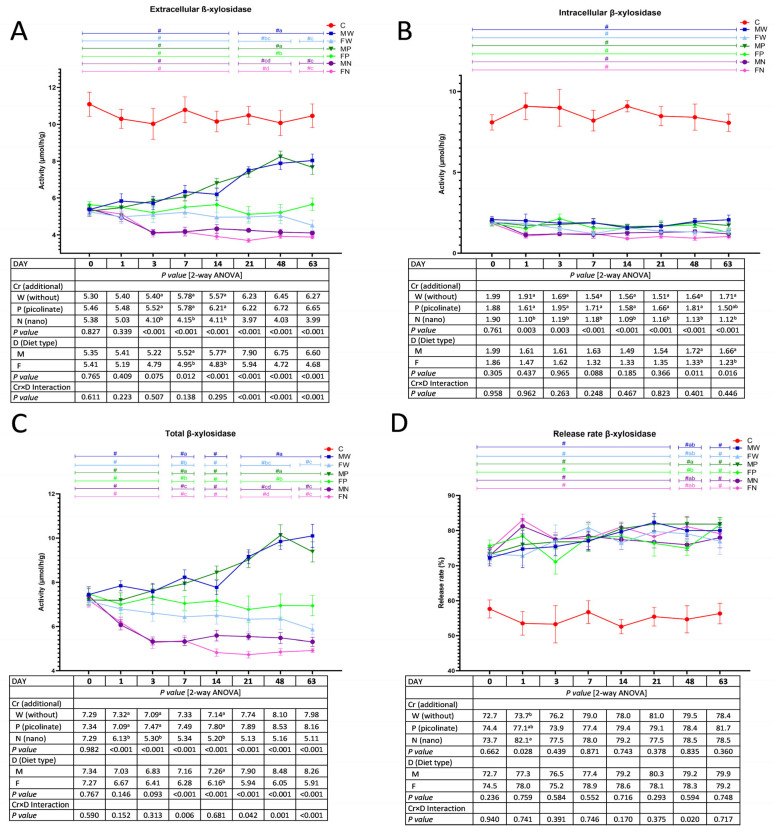
The extracellular (**A**), intracellular (**B**), total (**C**) activity and release rate (**D**) of faecal bacterial β-xylosidase during the experimental feeding period in rats (n = 8 per group). The details of the feeding periods are described in Table 1. C, animals fed a standard diet for laboratory rodents for 18 weeks; MW, animals fed the high-fat diet for 9 weeks and then the C diet for 9 weeks; FW, animals fed the high-fat diet for 18 weeks; MP, animals fed the high-fat diet for 9 weeks and then the C diet for 9 weeks with the addition of chromium picolinate; FP, animals fed the high-fat diet for 9 weeks and then the high-fat diet for 9 weeks with the addition of chromium picolinate; MN, animals fed the high-fat diet for 9 weeks and then the C diet for 9 weeks with the addition of chromium nanoparticles; FN, animals fed the high-fat diet for 9 weeks and then the high-fat diet for 9 weeks with the addition of chromium nanoparticles; M, all animals fed the high-fat diet for 9 weeks and then the C diet for 9 weeks, regardless of the type of chromium in the diet; F, all animals fed the high-fat diet for 18 weeks, regardless of the type of chromium in the diet. a–d mean values within a time point with unlike letters in the upper part of the figure are significantly different (*p* < 0.05). Only in the case of a statistically significant Cr×D interaction are the differences between groups highlighted with letters in the figure (two-way ANOVA, *p* < 0.05). # indicates a significant difference vs. the C group (*t* test, *p* < 0.05).

Two-way ANOVA showed that, irrespective of the diet type, the dietary application of chromium nanoparticles caused a significant decrease in the extracellular activity of microbial β-xylosidase in the rat faeces excreted on Days 3, 7, and 14 (*p* < 0.05 vs. the W and P treatments; Figure 4A). Regardless of the Cr addition, on Days 7 and 14, the M treatment enhanced faecal extracellular β-xylosidase activity compared to that with the F treatment (*p* < 0.05). The highest extracellular activity of the enzyme on Days 21, 48, and 63 was observed in the MW and MP groups (*p* < 0.05 vs. all other groups; see significant Cr×D interaction). The lowest extracellular β-xylosidase activity on Days 21 and 48 was in the faeces excreted by FN rats (*p* < 0.05 vs. MW, FW, MP, and FP), while the lowest activity on Day 63 was observed in the FW, MN, and FN groups (*p* < 0.05 vs. MW, MP, and FP). The *t* test showed that during the experimental weeks (10–18 weeks), all experimental rat faeces were characterised by lower extracellular, intracellular and total β-xylosidase activities in comparison to those in the control C group (*p* < 0.05; Figure 4B,C). Two-way ANOVA showed an increase in intracellular β-xylosidase activity in the M treatment versus the F treatment on Days 48 and 63, irrespective of chromium supplementation (*p* < 0.05; Figure 4B). From Day 1 until the end of the experimental feeding, the lowest intracellular activity of faecal bacterial β-xylosidase was observed upon Cr-NP dietary addition (*p* < 0.05 vs. the W and P treatments on Days 1–48 and *p* < 0.05 vs. W treatment on Day 63). Regardless of the diet type, on Days 1, 3, and 14, the dietary application of Cr-NPs caused a significant decrease in the total activity of bacterial faecal β-xylosidase (*p* < 0.05 vs. the W and P treatments; Figure 4C). On Day 14, the faeces of the M treatment group had higher total β-xylosidase activity than that of the F treatment group, irrespective of Cr supplementation. As indicated by the Cr×D interaction, the highest total β-xylosidase activity on Days 7, 21, 48, and 63 was observed in the MW and MP groups, while the lowest activity was caused by Cr-NP addition. On Days 21–48, the total β-xylosidase activity of the FW faeces was comparable to that of the MN group, while on Day 63, it was comparable to that of the MN and FN groups. The *t* test revealed a significantly elevated β-xylosidase release rate in the faeces of all rats fed experimental diets during the entire 10–18-week period compared to that in the control C rats (Figure 4D). Regardless of the diet type, the dietary Cr-NPs enhanced the β-xylosidase release rate on Day 1 in relation to that in the W treatment (*p* < 0.05). The Cr×D interaction on Day 48 significantly differed in the β-xylosidase release rate percentage between the dietary counterparts fed diets with chromium picolinate (MP > FP; *p* < 0.05).

### 3.5. Faecal Short-Chain Fatty Acids

**Figure 5:**
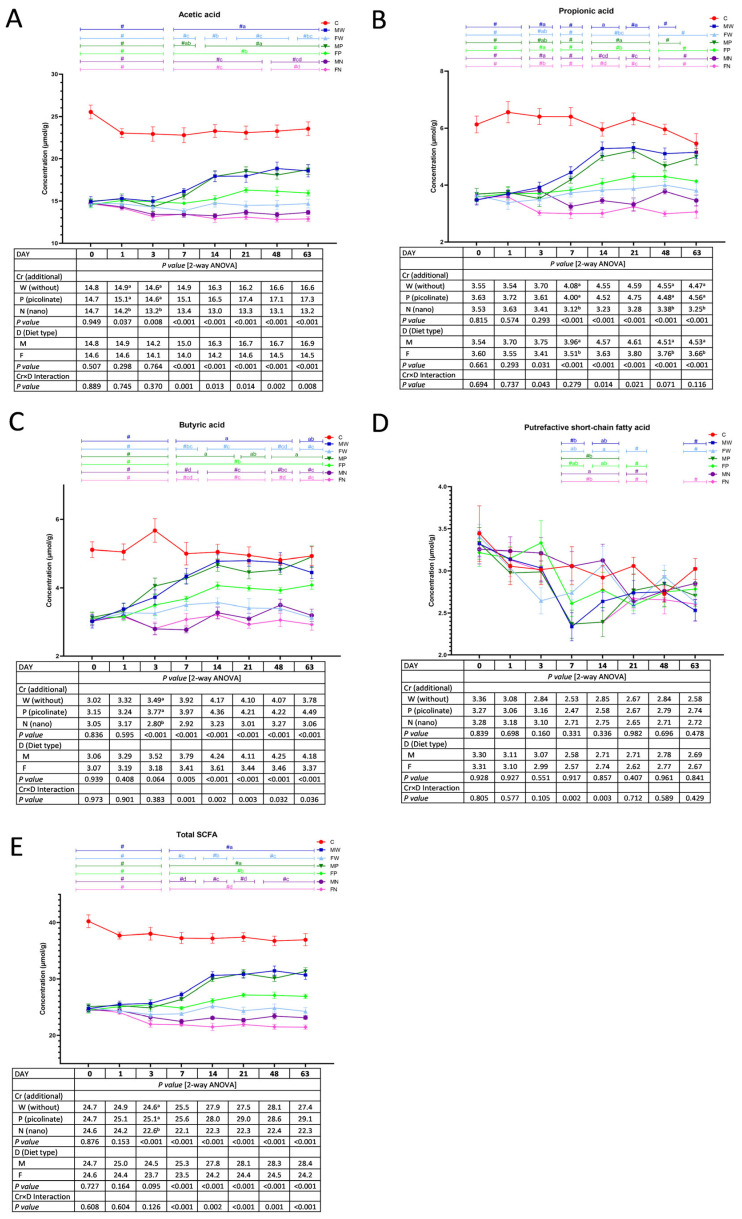
Faecal short-chain fatty acids during the experimental feeding period in rats (n = 8 per group). The details of the feeding periods are described in Table 1. C, animals fed a standard diet for laboratory rodents for 18 weeks; MW, animals fed the high-fat diet for 9 weeks and then the C diet for 9 weeks; FW, animals fed the high-fat diet for 18 weeks; MP, animals fed the high-fat diet for 9 weeks and then the C diet for 9 weeks with the addition of chromium picolinate; FP, animals fed the high-fat diet for 9 weeks and then the high-fat diet for 9 weeks with the addition of chromium picolinate; MN, animals fed the high-fat diet for 9 weeks and then the C diet for 9 weeks with the addition of chromium nanoparticles; FN, animals fed the high-fat diet for 9 weeks and then the high-fat diet for 9 weeks with the addition of chromium nanoparticles; M, all animals fed the high-fat diet for 9 weeks and then the C diet for 9 weeks, regardless of the type of chromium in the diet; F, all animals fed the high-fat diet for 18 weeks, regardless of the type of chromium in the diet. SCFA, short-chain fatty acids. a–d mean values within a time point with unlike letters in the upper part of the figure are significantly different (*p* < 0.05). Only in the case of a statistically significant Cr–D interaction are the differences between groups highlighted with letters in the figure (two-way ANOVA, *p* < 0.05). # indicates a significant difference vs. the C group (*t* test, *p* < 0.05). (**A**) Acetic acid; (**B**) Propionic acid; (**C**) Butyric acid; (**D**) Putrefactive SCFA (the sum of iso-butyric, iso-valeric and valeric acids); (**E**) Total short-chain fatty acids.

On Days 1 and 3, regardless of the diet type, the dietary addition of chromium nanoparticles caused a significant decrease in the faecal acetic acid concentration compared to that in the W treatments (without Cr) and with Cr-Pic (Figure 5A). The Cr×D interaction showed that from Day 7 to the end of the experimental period, the MW and MP groups had higher acetic acid concentrations in the faeces than those in the remaining groups (*p* < 0.05; except the MP and FP groups on Day 7). The lowest concentration of acetic acid was noted in the FN rats (*p* < 0.05 vs. MW, MP, and FP on Days 7 and 21; vs. MW, FW, MP, and FP on Days 14, 48 and 63). The *t* test revealed that during the entire experimental period (10–18 weeks), all groups were characterised by lower faecal acetic acid concentrations than those in the control C group (*p* < 0.05). As shown in Figure 5B, a significant Cr×D interaction was noted on Days 3, 14, and 21 with respect to the faecal concentration of propionic acid. On Day 3, the lowest C3 concentration was observed in the faeces excreted by FN rats (*p* < 0.05 vs. MW, FP, and MN). On Days 14 and 21, the highest concentration of propionic acid was found in the MW and MP groups (*p* < 0.05 vs. all other groups), while the lowest concentration was noted in the FN group (Day 14; *p* < 0.05 vs. all groups except MN) and the FN and MN animals (Day 21; *p* < 0.05 vs. MW, MP, and FP). The two-way ANOVA showed that on Days 7, 48, and 63, the dietary chromium nanoparticles decreased the faecal C3 content, regardless of the diet type, while the discontinuation of a high-fat diet increased the concentration, irrespective of Cr supplementation. Generally, compared to the control C group (*t* test), the faeces of the experimental groups showed diminished propionic acid concentrations during weeks 10–18, except for the MN and MP rats on Day 63. The faecal butyric acid (C4) concentration was decreased by dietary Cr-NP supplementation on Day 3, regardless of the diet type (*p* < 0.05 vs. the W and P treatments; Figure 5C). From Day 7 until the end of the experimental period, the Cr×D interaction was observed in relation to C4 acid. On those days, compared to the FW rats (fed the same high-fat diet as all experimental rats in the introductory period), enhanced butyric acid concentrations were found in the faeces of the MW, MP, and FP groups (except for Day 7 in the case of the latter group). The *t* test showed decreased butyric acid concentrations in the faeces of rats during the 10–18-week period versus the control group, except for the MW and MP rats on Days 7 to 63. The highest faecal concentration of PSCFAs on Day 7 was found in the MN group (*p* < 0.05 vs. MW, MP, and FN; Figure 5D) and in the FW and MN groups on Day 14 (*p* < 0.05 vs. MP and FN; see Cr×D interaction). Regardless of the diet type, dietary supplementation with Cr-NPs caused a decrease in the faecal total SCFA concentration on Day 3 (*p* < 0.05 vs. the W and P treatments; Figure 5E). A significant Cr×D interaction regarding the total faecal SCFA concentration was observed on Days 7–63. Compared to the FW rats, a significantly higher SCFA level was observed in the MW and MP groups (Days 7, 14, 21, 48, and 63) as well as in the FP animals (Days 7, 21, 48, and 63). The lowest faecal SCFA concentration was noted in rats fed diets supplemented with Cr-NPs (*p* < 0.05; MN and FN vs. all other groups on Days 7, 14, 21 and FN vs. the remaining groups on Days 48 and 63). The *t* test indicated that, during the entire experimental period (10–18 weeks), all groups were characterised by lower total faecal SCFA levels in comparison to those in the control C group (*p* < 0.05).

### 3.6. Faecal pH

**Figure 6:**
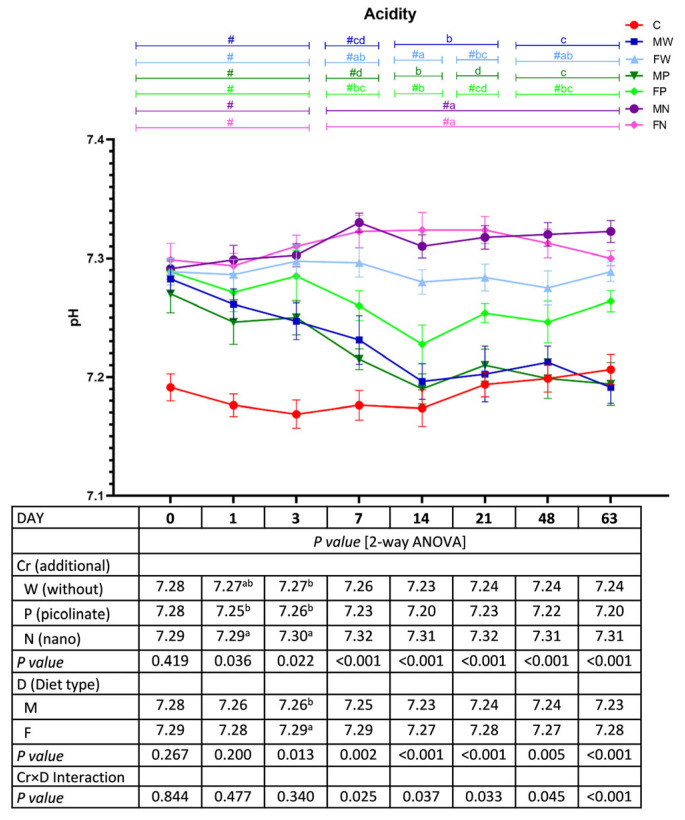
Faecal pH during the experimental feeding period in rats (n = 8 per group). The details of the feeding periods are described in Table 1. C, animals fed a standard diet for laboratory rodents for 18 weeks; MW, animals fed the high-fat diet for 9 weeks and then the C diet for 9 weeks; FW, animals fed the high-fat diet for 18 weeks; MP, animals fed the high-fat diet for 9 weeks and then the C diet for 9 weeks with the addition of chromium picolinate; FP, animals fed the high-fat diet for 9 weeks and then the high-fat diet for 9 weeks with the addition of chromium picolinate; MN, animals fed the high-fat diet for 9 weeks and then the C diet for 9 weeks with the addition of chromium nanoparticles; FN, animals fed the high-fat diet for 9 weeks and then the high-fat diet for 9 weeks with the addition of chromium nanoparticles; M, all animals fed the high-fat diet for 9 weeks and then the C diet for 9 weeks, regardless of the type of chromium in the diet; F, all animals fed the high-fat diet for 18 weeks, regardless of the type of chromium in the diet. a–d mean values within a time point with unlike letters in the upper part of the figure are significantly different (*p* < 0.05). Only in the case of a statistically significant Cr–D interaction are the differences between groups highlighted with letters in the figure (two-way ANOVA, *p* < 0.05). # indicates a significant difference vs. the C group (*t* test, *p* < 0.05).

The addition of chromium nanoparticles to the diet resulted in an increase in faecal pH on Days 1 (versus the P treatment) and 3 (versus the W and P treatments), regardless of diet type (*p* < 0.05; Figure 6). The M treatment had a significantly lower faecal pH than that with the F treatment on Day 3, irrespective of the Cr addition to the diet. From Day 7 until the end of the study, a significant Cr×D interaction was observed. On Day 7, the lowest faecal pH was observed in the MP rats (*p* < 0.05 vs. all groups except MW), and the highest values were noted in the faeces of MN and FN rats (*p* < 0.05 vs. all groups except FW). On Day 14, the FW, FN, and MN groups had higher faecal pH values than those in the other groups (*p* < 0.05). On Days 21, 48, and 63, the highest faecal pH was found in the MN and FN rats (*p* < 0.05 vs. all remaining rats), while the lowest faecal pH was noted in the MW and MP animals (*p* < 0.05 vs. FW, MN, and FN). The *t* test showed an increase in the faecal pH values during the experimental 10–18 weeks versus those in the control C group, except for the MW and MP rats from Day 14 to Day 63.

## 4. Discussion

The human and animal digestive tracts are colonised by a complex of billions of microorganisms. It is well known that the intestinal microbial community is responsible for the large intestinal fermentation of dietary oligosaccharides, polysaccharides and some proteins that escape digestion processes in the upper gastrointestinal tract [35]. The gut microbes can influence body weight, insulin sensitivity, or glucose and lipid metabolism; thus, some authors have reported that changes in the microbiota may be important in the pathogenesis of obesity and metabolic syndrome [36,37]. In the present study, we aimed to reveal the dynamics of the changes in the activity of selected bacterial enzymes and the amount of the most important products of bacterial metabolism in the large intestine, i.e., SCFAs, during several days of feeding rats a high-fat or standard-fat diet supplemented with two dietary Cr forms. The initial high-fat diet feeding of all rats made it possible to assess the changes in the parameters due to a change in dietary habits, i.e., from an unhealthy to a health-promoting diet. Some studies have shown that changing from a low-fat to a high-fat diet results in significant quantitative differences in the composition of the gut microbiota [38]. Taking this into account, the reverse situation, i.e., switching from a high-fat to a low-fat diet, would also bring about changes in the number and activity of the various bacterial groups.

Analyses of enzyme activity, including enzymes considered potentially harmful, as well as tests on the levels of bacterial metabolic products, e.g., SCFAs, are important because such molecules are the resulting “work” of the entire intestinal microbiota, and they interact locally in the gastrointestinal tract and, once absorbed, exert effects throughout the body [6,39,40]. In the present experiment, the results showed significant dynamism in the changes in enzymatic activity and the amount of SCFAs produced under the influence of switching to a diet with a standard fat and fibre content (standard diet), as well as under the influence of supplementation with chromium nanoparticles. In the first case, concerning the change from a high-fat to a low-fat diet, the change in enzymatic activity occurred rapidly, usually after the first day of the experimental dietary change, while a noticeable change in faecal SCFA concentrations occurred after seven days. The addition of chromium nanoparticles to the diet resulted in a very rapid metabolic response of the microbiota by reducing enzymatic activity and SCFA levels after only the first day of the experimental period. In contrast to nanoparticles, the addition of chromium picolinate to the diet had little, if any, effect on the aforementioned faecal parameters. Such a rapid change in the activity of the bacterial enzymatic apparatus in the face of new dietary components or changes in their quantity in the diet clearly indicates the tremendous adaptive power of intestinal microbiota [41]. It is very interesting to observe the variation in changes in extracellular and total activity, the latter including the former and intracellular activity, in the setting of a high-fat diet (i.e., without switching to a low-fat diet) or in the setting of supplementation with chromium nanoparticles. Regardless of the variations in individual enzymes, remaining on a high-fat diet increased extracellular activity. Only in the case of β-xylosidase were both the extracellular and the total activities reduced in the treatment with a high-fat diet. This was probably because the high-fat diet was also a low-fibre diet, and the aforementioned enzyme is responsible for the breakdown of complex polysaccharides, i.e., xylans and xylooligosaccharides [14]. In our previous experiments in rats fed a high-fat diet, we also found an increase in the extracellular activity of faecal β-glucosidase and β-glucuronidase, as well as enzymes analysed in the caecal contents of the rats, with an increase in the enzyme release rate from bacterial cells to the external environment [30,42]. The extracellular enzymatic activity of bacteria is important because it determines the scale and intensity of the metabolic processes for which the enzymes are predestined. Increased bacterial β-glucuronidase activity is not beneficial to the host intestinal ecosystem, as it is characteristic of the activity of strictly pathogenic and opportunistic bacteria [7]. Bacterial β-glucosidase activity is widespread among the microbiota, and it is responsible for releasing a wide range of plant secondary metabolites from their β-D-glucosylated precursors, which may have beneficial or harmful properties [43]. In the experiment presented here, the dietary addition of Cr-NPs inhibited the extracellular and total activity of the bacterial faecal enzymes, i.e., α-glucosidase, β-glucosidase, β-glucuronidase, and β-xylosidase. Interestingly, the release rate of the enzymes β-glucosidase and β-glucuronidase was, as with the high-fat diet treatment, significantly increased. A decrease in enzymatic activity following the addition of nanoparticles to the diet is not unusual, given the known antibacterial properties of various metal nanoparticles [44,45]. As indicated by an intriguing study by Ramin and Allison [6], the extracellular enzymatic activity of the microbiota can be increased both when nutrients are fully available to the bacteria and during their absence. The population of the gut microbiota can adapt in different ways to the prevailing environmental conditions to develop or survive as efficiently as possible. When there is a large amount of undigested substances flowing with the intestinal contents into the terminal gastrointestinal tract segments, bacterial enzymes are released from the cells into the external environment to make the best use of these substances, to produce as many metabolites as possible and, as a result, to optimally proliferate the entire microbiota population. In such cases, the percentage of extracellular enzymatic activity in relation to the total activity may be enhanced due to the large number of microorganisms themselves, and to their high metabolic activity, caused by the abundance of nutrients. Indeed, a line of research has pointed at extensive microbial carbohydrate metabolism as an additional energy-bringing element in the pathology of excess metabolic energy leading to a state of obesity and prediabetes [46,47]. In our trial, such an effect was partially observed in the treatments with a high-fat diet. This was in part because the high-fat diet was also low in fibre, and dietary fibre is the best source of potential energy for the microbiota. The situation is different in the presence of antimicrobial substances, e.g., metal nanoparticles, or a low nutrient content in the intestinal digesta. The microbial population is reduced, and the bacteria release as many enzymes as possible into the environment to make the most efficient use of what they can from the low substrate supply, allowing them to survive under unfavourable conditions [48]. These different adaptive mechanisms may therefore lead to a similar increase in the percentage of extracellular enzyme activity in relation to the total activity, as in our study, with regard to a high-fat diet and chromium nanoparticle supplementation.

Interestingly, both the high-fat diet and the dietary addition of chromium nanoparticles resulted in a significant reduction in SCFA concentrations in the rat faeces during the experiment. The faecal SCFA reduction upon dietary nanoparticles could be ascribed to the potential antimicrobial properties of Cr-NPs, the components of which are both a quantitative reduction in the number of bacterial populations as well as a reduction in the enzymatic potential of the intestinal microbiota. The scientific literature describing studies on the effects of chromium nanoparticles on the gut microbiota is not extensive, unlike that relating to Ag, Cu, Se, TiO_2_, and Zn nanoparticles [49,50,51,52]. Therefore, it can be considered that the research in this study fills an existing gap in our knowledge. In a mouse study by Yang and co-authors [53], the inorganic addition of chromium to the diet had no effect on the caecal microbiota, unlike a supplement combining both Cr and a probiotic. A similar effect was observed by Guo et al. [54] in STZ-induced diabetic mice fed a high-fat diet when Cr(III) was combined with *Grifola frondosa* polysaccharide. The studies presented here indicate that chromium picolinate has little effect on the metabolic activity of the faecal microbiota, as opposed to the nanoparticles of this element. This finding is confirmed by our previous studies on the effects of various forms of chromium on the microbiota of the rat caecum [55]. The antibacterial properties of ingested nanoparticles are described as their ability to disrupt the bacterial cell wall and/or to generate reactive oxygen species, which can also impair biostructures, e.g., cell-wall components and DNA [44,45]. Without an efficiently and effectively working enzymatic apparatus, the microbiota is unable to produce significant amounts of SCFAs [56,57]. It is understandable that a measurable reduction in body weight under the influence of a change in diet takes time, while changes in the metabolic activity of the microbiota are visible much earlier. In a well-designed study by Ruiz et al. [58], only long-term (one year) restriction in caloric intake resulted in significant and lasting changes in the genome of bacteria populating the large intestine of patients struggling with obesity. It should also be stressed that the decrease in the faecal SCFA concentrations caused by the dietary application of Cr-NPs was observed both in high-fat and low-fat dietary regimens. As a result, an increase in faecal pH values created a less favourable environment for optimal beneficial bacterial growth. Another observation found during experimental feeding was that there was no change in the amount of PSCFAs produced, with a decrease in total SCFAs, under the influence of the high-fat diet and chromium nanoparticles. Valerate, isovalerate and isobutyrate constitute the PSCFAs, and those acids are produced exclusively by protein fermentation. These SCFAs are putrefactive and suggest underlying protein maldigestion or malabsorption under the high-fat dietary regimen and under the dietary application of chromium nanoparticles. Dietary protein that is not digested or absorbed in the small intestine may be fermented by colonic bacteria to produce products of protein breakdown, e.g., PSCFAs and ammonia.

## 5. Conclusions

The present study showed that under undesirable dietary conditions, in this case a high-fat, low-fibre diet, as well as under changes in dietary habits—in this study, switching from a high-fat to a low-fat diet—the faecal microbial metabolic activity showed different adaptation mechanisms. These mechanisms involved an increase in bacterial enzymatic extracellular activity, lowered intracellular activity, and an enhanced rate of enzyme secretion from bacterial cells into the faecal environment. Dietary supplementation with chromium nanoparticles further modulated the aforementioned microbial activity, i.e., diminished the extracellular and total enzymatic activities, while the effect of chromium picolinate addition was rather negligible. Both the high-fat diet and the addition of chromium nanoparticles reduced the SCFA concentrations and increased the faecal pH values. Comparing the dietary factors used, it can be stated that the beneficial health-promoting effects on the metabolic activity of the microbiota of obese rats are related to a change in dietary habits by switching from a high-fat diet to a low-fat diet.

## Figures and Tables

**Table 1:** Schema of the dietary treatments applied to rats during the introductory and experimental periods.

Group	Control C	MW	FW	MP	FP	MN	FN
Introductory period (1–9 weeks)	Diet C	Diet F					
Cr dietary addition	Without Cr						
Experimental period (10–18 weeks)	Diet C		Diet F	Diet C	Diet F	Diet C	Diet F
Cr dietary addition *	Without Cr			Cr-Pic		Cr-NP	

* daily additional Cr dose 0.3 mg/kg body weight; Cr-Pic, chromium picolinate; Cr-NPs, chromium nanoparticles. Low-fat C diet (g/100 g): casein 14.8; DL-methionine 0.20; cellulose 8.0; choline chloride 0.20; cholesterol 0.30; vitamin mix 1.0; mineral mix 3.5; rapeseed oil 8.0; maize starch 64.0. High-fat F diet (g/100 g): casein 14.8; DL-methionine 0.20; cellulose 3.0; choline chloride 0.20; cholesterol 0.30; vitamin mix 1.0; mineral mix 3.5; rapeseed oil 8.0; lard 17.0; maize starch 52.0.

## Data Availability

Data supporting the reported results are available on request.
